# PotatoBSLnc: a curated repository of potato long noncoding RNAs in response to biotic stress

**DOI:** 10.1093/database/baaf015

**Published:** 2025-02-22

**Authors:** Pingping Huang, Weilin Cao, Zhaojun Li, Qingshuai Chen, Guangchao Wang, Bailing Zhou, Jihua Wang

**Affiliations:** Shandong Provincial Key Laboratory of Biophysics, Institute of Biophysics, Dezhou University, No. 566 Daxue West Road, Decheng District, Dezhou 253023, China; The Engineering Research Institute of Agriculture and Forestry, Ludong University, No. 186 Hongqi Middle Road, Zhifu District, Yantai 264025, China; College of Computer and Information Engineering, Dezhou University, No. 566 Daxue West Road, Decheng District, Dezhou 253023, China; Shandong Provincial Key Laboratory of Biophysics, Institute of Biophysics, Dezhou University, No. 566 Daxue West Road, Decheng District, Dezhou 253023, China; College of Computer and Information Engineering, Dezhou University, No. 566 Daxue West Road, Decheng District, Dezhou 253023, China; Shandong Provincial Key Laboratory of Biophysics, Institute of Biophysics, Dezhou University, No. 566 Daxue West Road, Decheng District, Dezhou 253023, China; Shandong Provincial Key Laboratory of Biophysics, Institute of Biophysics, Dezhou University, No. 566 Daxue West Road, Decheng District, Dezhou 253023, China

## Abstract

The biotic stress significantly influences the production of potato (*Solanum tuberosum* L.) all over the world. Long noncoding RNAs (lncRNAs) play key roles in the plant response to environmental stressors. However, their roles in potato resistance to pathogens, insects, and other biotic stress are still unclear. The PotatoBSLnc is a database for the study of potato lncRNAs in response to major biotic stress. Here, we collected 364 RNA sequencing (RNA-seq) data derived from 12 kinds of biotic stresses in 26 cultivars and wild potatoes. PotatoBSLnc currently contains 18 636 lncRNAs and 44 263 mRNAs. In addition, to select the functional lncRNAs and mRNAs under different stresses, the differential expression analyses and the Gene Ontology (GO) and Kyoto Encyclopedia of Genes and Genomes (KEGG) analyses related to the *cis*/*trans*-targets of differentially expressed lncRNAs (DElncRNAs) and to the differentially expressed mRNAs (DEmRNAs) were also conducted. The database contains five modules: Home, Browse, Expression, Biotic stress, and Download. Among these, the “Browse” module can be used to search detailed information about RNA-seq data (disease, cultivator, organ types, treatment of samples, and others), the exon numbers, length, location, and sequence of each lncRNA/mRNA. The “Expression” module can be used to search the transcripts per million/raw count value of lncRNAs/mRNAs at different RNA-seq data. The “Biotic stress” module shows the results of differential expression analyses under each of the 12 biotic stresses, the *cis*/*trans*-targets of DElncRNAs, the GO and KEGG analysis results of DEmRNAs, and the targets of DElncRNAs. The PotatoBSLnc platform provides researchers with detailed information on potato lncRNAs and mRNAs under biotic stress, which can speed up the breeding of resistant varieties based on the molecular methods.

**Database URL**: https://www.sdklab-biophysics-dzu.net/PotatoBSLnc

## Introduction

The potato (*Solanum tuberosum* L.) has gradually become the fourth most important staple crop worldwide after wheat, corn, and rice, since it produces tubers of high quantity and nutritional quality [1]. However, multiple biotic stresses, including pathogens and insects, might severely devastate the production and quality of potatoes [2]. Late blight caused by *Phytophthora infestans* is one of the most important potato diseases, which leads to huge economic losses every year [3]. Furthermore, cyst nematodes, including *Globodera rostochiensis*, and *Globodera pallida*, occur in potato-growing areas in >75 countries recently and also cause significant economic losses [4]. The Colorado potato beetle feeds on leaves of potato plants and therefore might limit crop growth and reduce tuber yields [5].

Biotic stresses are major factors that influence potato yields. To control the influence of biotic stresses, breeding resistant varieties based on the molecular methods are more effective in contrast with the conventional breeding methods. Recent with transcriptome sequencing allow us to acquire a comprehensive understanding of key biological processes to identify genes or transcription factors (TFs) underlying traits like disease stresses [6]. For example, the nine functional TFs in defensive responses in potatoes were found, such as auxin response factors, zinc-finger protein, and no apical meristem, Arabidopsis thaliana activating factor1/2, and cup-shaped cotyledon (NAC) [7]. The upregulated genes, including heat shock protein, terpene synthase, and kinase encoding genes, indicated their potential roles in *Potato virus Y* (PVY) infection based on RNA sequencing (RNA-seq) [8]. Furthermore, the differentially expressed genes mainly enriched in the salicylic acid biosynthesis, systemic acquired resistance, and calcium-binding activity pathways based on the RNA-seq in potatoes infected by potato cyst nematode and control samples [9]. In addition to the mRNAs and TFs, long noncoding RNAs (lncRNAs) also play crucial roles in plants’ environmental stress tolerance, including abiotic and biotic stresses.

LncRNAs are transcripts with a length of >200 nt and lack coding ability, and they can regulate gene expression at different levels and therefore participate in the regulation of almost all biological processes [10–12]. The previous studies mainly focus on the function of lncRNAs in plant abiotic tolerance [13]. LncRNAs can control the drought, heat, cold, salinity, nutrient deficiency, and heavy metal toxicity stress tolerance in multiple plant species, like *Arabidopsis, Brassica juncea*, and Wheat [14, 15]. Differently, the functional role of lncRNAs in various plants’ biotic stress tolerance at multiple regulatory levels is an emerging field and is gradually becoming a hot spot. For example, the lncRNA *SABC1* can repress salicylic acid production and plant immunity via decreasing its neighboring gene (*NAC3*) and isochorismate synthase 1 transcription, and the downregulation of it can derepress plant resistance to bacteria and viruses upon pathogen infections [16]. A recent study constructed the *lncRNA14234-miR394a-5p-SPL11* regulatory network in maize-resistant and susceptible lines responsive to sugarcane mosaic virus (SCMV) and found that silencing *lncRNA14234* could decrease the accumulations of SCMV and SPL11 targeted by *miR394a-5p* [17]. The tomato *lncRNA39896* could negatively regulate resistance to *P. infestans* by the *lncRNA39896-miR166b-HDZs* module [18]. In addition to the bacteria, fungal, and virus stresses, lncRNAs also involve in the regulation of other plant biotic stresses, including nematodes and insects. A total of 4453 differentially expressed lncRNAs (DElncRNAs) were identified among the infected and noninfected peanuts of root-knot nematodes, and some of them could regulate the oxidation–reduction process [19]. A total of 15 and 6 lncRNAs were discovered, which might participate in the response to soybean cyst nematode (*Heterodera glycines*) and *Rotylenchulus reniformis* infection in soybean [20]. Furthermore, the cotton *lncA07* and *lncD09* played key roles in whitefly aphid resistance in cotton [21]. However, despite some functional lncRNAs in various plant species biotic stress tolerance has been discovered, and the research about potato lncRNAs related to resistance regulatory mechanisms against biotic stresses is still in its infancy. To date, only potato lncRNAs responsive to *Pectobacterium carotovorum* and *P. infestans* infections have been revealed [2, 22, 23], the potato lncRNAs related to other biotic stress tolerance are still unknown.

Over the last decades, with the development of RNA-seq methods, more and more plant RNA-seq data have been obtained and some databases for plant lncRNAs have been built based on public plant RNA-seq datasets. They are CANTATAdb v3.0 with 8440 potato lncRNAs from 27 RNA-seq data [24], GreeNC with 8005 potato lncRNAs [25], NONCODE with 3069 potato lncRNAs [26], and PLncDB with 16 485 potato lncRNAs from 441 RNA-seq data [27]. All of these databases have greatly facilitated the plant lncRNA studies and include potato lncRNAs, but they lack potato biotic stress-related lncRNAs. Some databases contain plant lncRNAs related to abiotic and biotic stresses, including plant stress RNA-seq Nexus (PSRN) with 26 plant-stress RNA-seq datasets, but the potato is not included [28]; JustRNA with 3692 RNA-seq datasets obtained from 825 abiotic/biotic stresses and various hormone treatments in six model plants, but the potato is not included [29]; PncStress with six PVY-related lncRNAs in potato [30], and EVLncRNAs 3.0 with one experimentally validated *P. infestans*-related potato lncRNA [31]. Thus, it is highly desirable to have a platform to explore the biotic stress-related lncRNAs in potatoes.

**Figure 1.**
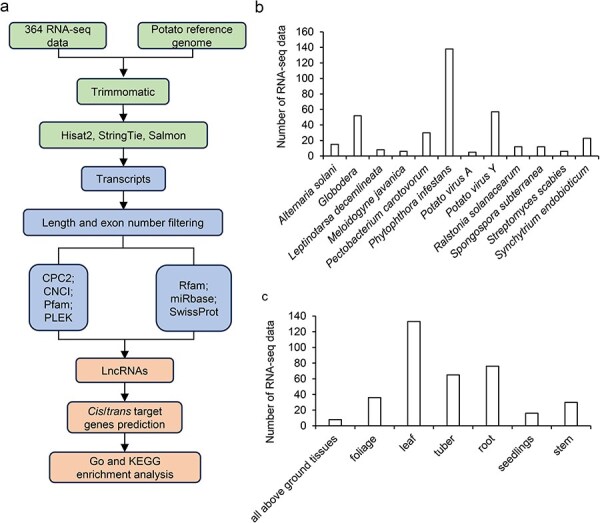
Overview of the potato lncRNA annotation and sample information. (a) The pipeline for the identification of lncRNAs. (b) The number of RNA-seq data at different disease/biotic stresses. (c) The number of RNA-seq data at different organ types.

This study developed a database of potato lncRNAs’ expression profiles in response to biotic stress according to the standard pipeline (Fig. 1a), named PotatoBSLnc. The platform contains: (i) the annotations of 18 636 lncRNAs and 44 263 mRNAs, like the location, exon numbers, and sequences; (ii) the expression profiles of each lncRNA and mRNA in the 364 RNA-seq data under 12 kinds of biotic stresses; and (iii) the results of differential expression analyses under specific biotic stress, the *cis*/*trans*-targets of DElncRNAs, the Gene Ontology (GO) and Kyoto Encyclopedia of Genes and Genomes (KEGG) analysis results of the differentially expressed mRNAs (DEmRNAs), and the targets of DElncRNAs.

## Materials and methods

### RNA-seq data collection

We first identified articles related to potatoes from the PubMed database that were connected with transcriptome (up to 31 December 2022). To comprehensively obtain the potato transcriptome datasets, we used a list of keywords containing all the synonyms of transcriptome during the query, such as “RNA-seq,” “RNA sequencing,” “transcriptomics,” “transcriptomic,” “Illumina sequencing,” and “high-throughput sequencing.” Then, we manually verified the detailed information in each article, including the treatment of samples used for RNA extraction and sequencing, organ types, development stage, cultivar, and disease/biotic stress. Datasets with no biotic stress information and with special sequencing methods, like single-ended sequencing, were excluded. Only datasets with biotic stress information and paired-end sequencing were downloaded for further analysis. In total, 364 RNA-seq data derived from 12 kinds of biotic stresses in 26 cultivars and wild potatoes were obtained from the National Center for Biotechnology Information (NCBI), Genome Sequence Archive (GSA), and European Bioinformatics Institute (EBI) databases.

### Data analysis pipelines

#### Data preprocessing

The original compressed files were converted into fastq format by using the fastq-dump function in the SRA Toolkit (v 3.0.1). The adaptors and low-quality sequences with AVGQUAL <30 were trimmed by the trimmomatic software (v.0.39) to obtain the clean data [32].

#### LncRNA identification

The clean data were aligned to the potato reference genome (DM 1-3 516 R44; http://spuddb.uga.edu/data/DM_13_516_R44_potato_genome_assembly.v6.1.fa.gz) released in 2020 [33] by using the hisat2 software (v 2.1.0) [34]. On average, 76.32% of the reads were successfully aligned with the reference genome. We assembled the transcripts of each transcriptome sample separately by StringTie (v2.1.3), and all the 364 gtf files were then merged into one using the merge function in StringTie [34] (Fig. 1a). We then compared the assembled transcript with the potato genome annotation information by using gffcompare software (v 0.12.6).

The transcripts with exon number higher than one and length >200 bp were retained for identification of lncRNAs. There are many software tools available for coding ability prediction of transcripts, such as CNCI [35], CPAT [36], CPC2 [37], DeepPlnc [38], Pfam [39], PLncPro [40], and PLEK [41]. In this study, to improve the accuracy of results, we combined four tools to calculate the coding potential of transcripts, including CPC2, CNCI, Pfam, and PLEK. Furthermore, the transcripts with an open reading frame length >120 amino acids were removed. To filter rRNA, tRNA, sRNA, and miRNA from lncRNAs, we compared the transcripts with the Rfam database (release 14.9) based on cmscan (Infernal v 1.1.4). Then, the blastn was used to filter the transcripts overlapping all mature miRNA sequences in the miRbase with the parameter *E*-value <1 × 10^–5^ [42]. Moreover, we used the blastx program to compare the sequences against SwissProt (2023/10) to discard the transcripts that might encode short peptides with the parameter -e 1.0e-4. Finally, only the transcripts that satisfied all the criteria were considered as lncRNAs in this database.

#### Differential expression analysis

We used the Salmon (v 1.10.1) software to quantify the count and transcripts per million (TPM) value of all assembled transcripts at different RNA-seq data [43]. The differential expression of transcripts in selected samples with replicates was calculated by the DESeq2, while the selected samples with no replicate were calculated by the edgeR packages in R (v 4.0.3) [44, 45]. The transcripts with *P*_adj_ ≤ .05 and |log_2_(fold change)| > 1 were defined as differentially expressed transcripts.

#### Target prediction

The DEmRNAs within 100 kb 5ʹ upstream or 3ʹ downstream of each DElncRNA were identified as potential *cis*-targets [2]. The DEmRNAs with a Pearson correlation coefficient of |*r*| ≥ 0.98 and *P* ≤ .05 to DElncRNA were considered as potential *trans* targets. The Pearson correlation between the DElncRNAs and the DEmRNAs expression patterns was calculated using R (v 4.0.3). To understand the function of DElncRNAs and DEmRNAs, the KEGG and GO enrichment analysis based on the DEmRNAs and the targets of DElncRNAs were performed using TBtools (v 2.028) [46].

#### Database construction

We built the backend using Spring Boot and MyBatis and designed the frontend with Vue 3, completing the functionality of various frontend and backend modules. Users can quickly browse the website interface through the browser. The entire system achieves secure and effective data communication between frontend and backend through axios and introduces Mapper layer data persistence technology. In conclusion, the Controller layer of the system encapsulates the processing results into Result and returns them to the frontend, completing the specific process of the frontend and backend interaction.

## Results

### Data content of PotatoBSLnc

Data in the PotatoBSLnc were based on 364 RNA-seq data derived from 12 kinds of biotic stresses in 26 potato cultivars and wild potatoes (Table 1). The 12 biotic stresses included *Alternaria solani*, *Globodera* spp., *Leptinotarsa decemlineata* (*L. decemlineata*), *Meloidogyne javanica*, *P. carotovorum, P. infestans, Potato virus A* (PVA), PVY, *Ralstonia solanacearum*, *Spongospora subterranean*, *Streptomyces scabies*, and *Synchytrium endobioticum*. Among all the 364 RNA-seq data, the number of RNA-seq data belonging to the *P. infestans*-infected potatoes and related control samples was the highest with 138 data, followed by that belonging to the PVY- and *Globodera* spp.-infected and control samples (Fig. 1b). The organ types used for RNA-seq included all above ground tissues, foliage, leaf, tuber, root, seedlings, and stem (Fig. 1c). Using a standard pipeline for the data analysis, 18 636 lncRNAs and 44 263 mRNAs were discovered.

**Table 1.** Summary of all RNA-seq data in PotatoBSLnc

Repository/	Number of RNA-seq data	Cultivar	Disease/biotic stress	Organ types	References
Accession number					
NCBI/PRJNA803348	23	Qingshu 9	*S. endobioticum*	Tuber	[47]
NCBI/PRJNA755645	15	Désirée	*A. solani*	Leaf	[48]
NCBI/PRJNA635213	18	Helan 15	*R. solanacearum; P. infestans*; PVY	Leaf	[49]
NCBI/PRJNA532699	6	Green Mountain; Hindenburg	*S. scabies*	Tuber	[50]
NCBI/PRJNA371556	5	A6	PVA	Leaf	[51]
NCBI/PRJNA768797	24	Russet Burbank; Payette Russet	PVY	Leaf	[8]
NCBI/PRJNA488526	4	Kufri Jyoti; Kufri Swarna	*Globodera* spp.	Root	[52]
NCBI/PRJNA754031	12	E-potato-3; E-potato-3 Pi04089 transgenic lines	*P. infestans*	Leaf	[53]
NCBI/GSE74871	30	BP1; Valor	*P. carotovorum*	Stem	[54]
NCBI/GSE142002	27	NahG-Rywal; Rywal; shRBOHD transgenic lines	PVY	Leaf	[55]
NCBI/PRJNA203403	36	Russet Burbank; transgenic “Russet Burbank” line SP2211	*P. infestans*	Tuber	[56]
NCBI/PRJNA318049	36	Russet Burbank; transgenic “Russet Burbank” line SP2211	*P. infestans*	Foliage	[56]
GSA/CRA000806	16	Tetraploid potato genotype SD20	*P. infestans*	Seedlings	[57]
NCBI/PRJNA616420	24	*Solanum cardiophyllum; Solanum pinnatisectum*	*P. infestans*	Leaf	[58]
NCBI/PRJNA242936	8	Igor; Desiree coi1 transgenic plants; Desiree	*L. decemlineata*	Leaf	[59]
NCBI/PRJNA358831	6	*Solanum commersonii*	*R. solanacearum*	Root	[60]
NCBI/GSE134790	6	Mondial	*M. javanica*	Root	[61]
GSA/CRA001611	8	Desiree; Desiree transgenic line R1, R3a, R3b	*P. infestans*	all above-ground tissues	[62]
NCBI/PRJNA776331	12	Gladiator; Iwa	*S. subterranea*	Root	[63]
NCBI/PRJNA515801	30	*Solanum phureja* k-11,291; *Solanum phureja* k-9836	*Globodera rostochiensis*	Root	[64]
EBI/E-MTAB-11646	18	Potato genotype SH	*G. pallida*	Root	[9]

### LncRNA features of PotatoBSLnc

**Figure 2.**
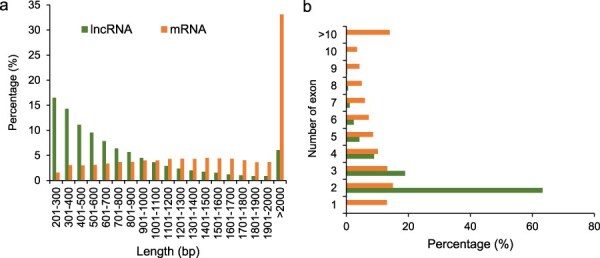
Characterization of potato lncRNAs. (a) Percentage of lncRNAs and mRNAs with different lengths. (b) Percentage of lncRNAs and mRNAs with different numbers of exons.

We compared the length and exon numbers between lncRNAs and mRNAs. The length of 75.9% lncRNAs ranged from 201 to 1000 bp, while that of 74.6% mRNAs were >1000 bp. The percentage of lncRNAs with length varied from 201 to 300 bp was the highest, which accounted for 16.5% of the total lncRNAs. The percentage of mRNAs with length >2000 bp was the highest, which accounted for 33.1% of the total mRNAs (Fig. 2a). The percentages of both the lncRNAs and mRNAs with two exons were the highest, which accounted for 63.3% and 15.0% of the total lncRNAs and mRNAs, respectively (Fig. 2b).

For a given lncRNA, PotatoBSLnc provides all basic information, including length, exon numbers, nucleotide sequence, and location. Furthermore, PotatoBSLnc provides expression profiles (raw count and TPM) for lncRNAs at each RNA-seq data under different biotic stresses.

### Stress-specific DElncRNAs analysis and function prediction

**Figure 3.**
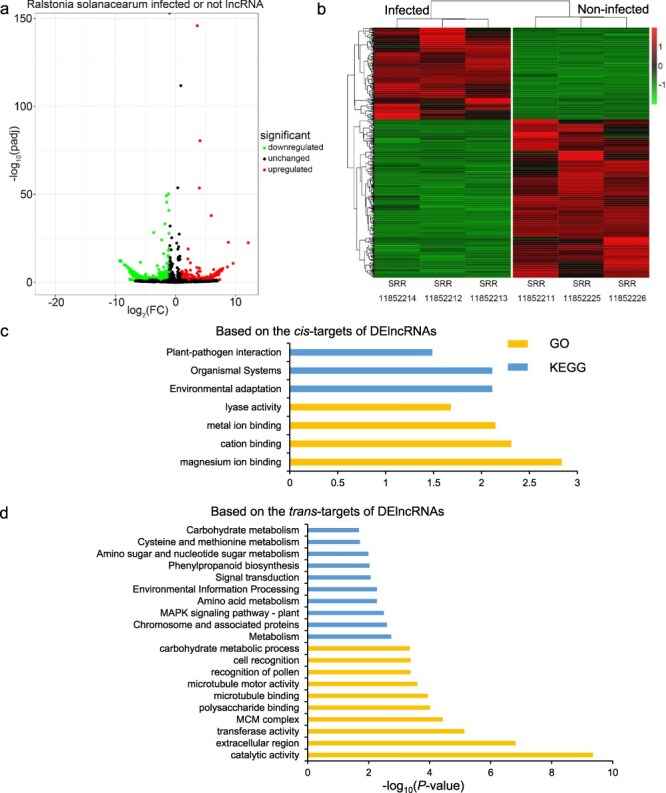
Analysis of lncRNAs from *R. solanacearum*-infected and -related control RNA-seq data. (a) Volcano map of DElncRNAs between the infected and noninfected groups of *R. solanacearum*. (b) Heat map of DElncRNAs. (c, d) The top 10 GO terms and KEGG pathways based on the *cis*/*trans* targets of DElncRNAs. The significant GO terms or KEGG pathways <10 showed all the significant terms or pathways.

For each of the 12 biotic stresses, we selected the subset of related RNA-seq data to identify the DElncRNAs among infected and control samples. Then, the GO and KEGG analyses based on the *cis*- and *trans*-targets of DElncRNAs were performed to help understand the functional roles of potato lncRNAs in response to each biotic stress. For example, samples SRR11852212, SRR11852213, and SRR11852214 were infected by *R. solanacearum* for 2 days, while samples SRR11852211, SRR11852225, and SRR11852226 were related control group. The differential expression analyses among the *R. solanacearum*-infected and control groups were implemented, resulting in a total of 670 DElncRNAs (Fig. 3a). These specific DElncRNAs might play important roles in the potato response to *R. solanacearum* infection. The expression patterns of DElncRNAs in the two groups were greatly different (Fig. 3b). Furthermore, we investigated the function of these DElncRNAs based on the *cis*- and *trans*-targets of them by the GO and KEGG enrichment analysis, respectively (Fig. 3c and d). The results revealed significant enrichment of four GO terms, including the “lyase activity” term, and three KEGG pathways, such as “Environmental adaptation” and “Plant–pathogen interaction,” based on *cis*-targets. For *trans* targets, 150 GO terms, including the “transferase activity” term, and 19 KEGG pathways, such as “Phenylpropanoid biosynthesis” and “Flavonoid biosynthesis,” were significantly enriched.

### Functions of PotatoBSLnc

PotatoBSLnc provides a user-friendly web interface that integrates biotic stress-related RNA-seq data of potatoes. The interface of PotatoBSLnc allows users to browse all data and download.

#### Homepage

The homepage of the database contains seven sections. On the upper left of the homepage, users can quickly have a general insight into the database by “Overviews” section. On the upper right of the homepage, users can browse the usage of the database. In the central part of the homepage, quick access portals are provided for users to view the main contents of the database, including “Contents” and “Biotic stresses (lncRNA)” sections. The “Contents” section contains four gateways, including “Samples,” “LncRNA information,” “LncRNA sequence,” and “LncRNA expression.” The “Biotic stresses (lncRNA)” section contains 12 gateways, each of them represents an entry point to lncRNAs of particular biotic stress. The users can quickly access the results of differential expression analyses of lncRNA, the *cis*- and *trans*-targets of DElncRNAs, and the GO and KEGG analyses related to the targets of the DElncRNAs by clicking the corresponding gateway. Below the “Biotic stresses (lncRNA)” section, the homepage showcases some data statistics from the database, such as the number of the RNA-seq data among different biotic stresses or organ types. In the “Related Publications by Our Team” section, we display our papers published on lncRNA, and a more detailed description of this section can be accessed below. The “Publications Related to Potato lncRNAs” section presents images and links to publications related to potato lncRNA, and users can browse the articles by clicking the images.

#### Browse

**Figure 4.**
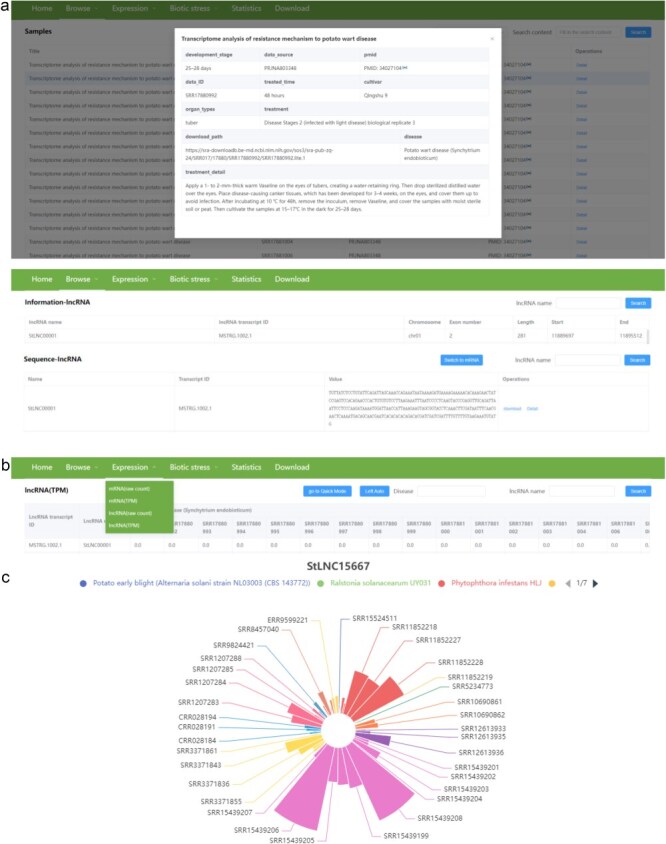
The user interface of the “Browse” and “Expression” contents in PotatoBSLnc. (a) The data displayed after clicking the “Samples,” “Information,” “Sequence” sub-menus in the “Browse” menu. (b) The “Expression” dropdown options. (c) The figure displayed after clicking the *StLNC15667* corresponding row in the lncRNA (TPM) page.

There are three pull-down sub-menus in the “Browse” menu, including samples, information, and sequence. The “Samples” sub-menu provides detailed information about the 364 RNA-seq data, including disease, cultivator, organ types, development stage, data source and ID, PMID of the corresponding article, download path, and treatment of samples. The “Information” sub-menu provides location (chromosome, start, and end site), exon number, and length of each lncRNA and mRNA. The “Sequence” sub-menu provides nucleotide sequence of each lncRNA and mRNA. Users can download the sequence of the interested lncRNA/mRNA in fasta format by clicking the download button in the operations column (Fig. 4a).

#### Expression

There are four pull-down sub-menus in the “Expression” menu, including mRNA (raw count), mRNA (TPM), lncRNA (raw count), and lncRNA (TPM) (Fig. 4b). Users can search the raw count and TPM values of each lncRNA/mRNA at 364 RNA-seq data. We also provide the visualization of expression patterns for lncRNA/mRNA. The lncRNA/mRNA expression patterns among different biotic stress samples will be directly shown in the figure after clicking the lncRNA name corresponding row, and the samples related to the same biotic stress have the same color. Notably, the different strains belonging to the same disease are also indicated by different colors. The size of the sector in the figure is determined by the expression level, and the sample with a larger sector represents a higher expression level of lncRNA/mRNA. For example, we search the *StLNC15667* in the lncRNA (TPM) page and click the lncRNA name corresponding row, and then the expression pattern of *StLNC15667* among all samples will be shown. From the figure, we can easily find that the expression of *StLNC15667* might correlate with the potato response to *P. infestans* infection (Fig. 4c). Furthermore, if the users are only interested in a certain disease or biotic stress, they can enter the corresponding disease in the Disease box and then clicking the search button, the expression profiles of lncRNA/mRNA at the related RNA-seq data will be shown. In addition, users can also browse the detailed information about the interested RNA-seq data by clicking the data ID in the expression page.

#### Biotic stress

**Figure 5.**
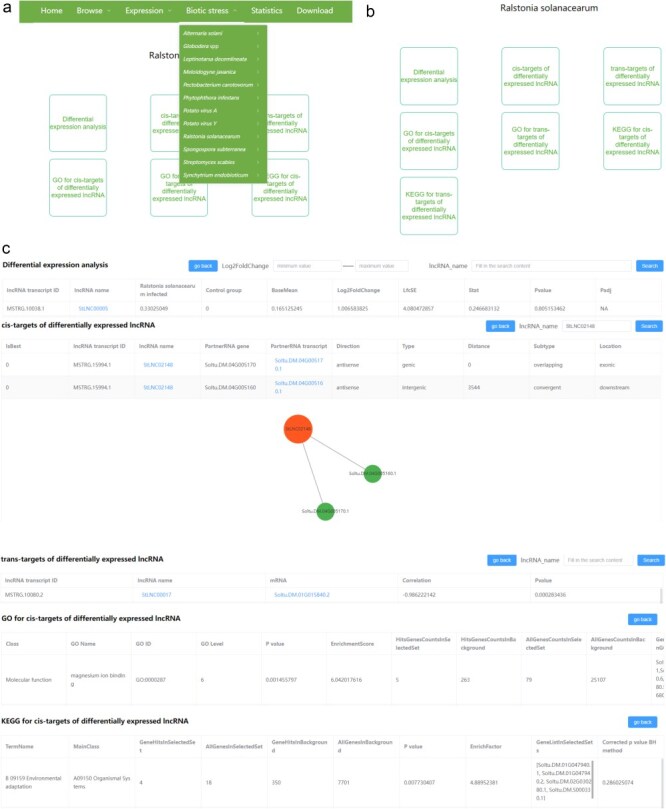
The user interface of the “Biotic stress” content in PotatoBSLnc. (a) The “Biotic stress” dropdown options. (b) The entries displayed after clicking each of the 12 biotic stress-lncRNA sub-menu. (c) The data displayed after clicking some entries in (b).

There are 12 pull-down sub-menus in the “Biotic stress” menu, each belonging to different disease types. Each sub-menu contains two parts, including lncRNA and mRNA (Fig. 5a). Users can browse the results of differential expression analyses of lncRNA and mRNA, the *cis*- and *trans*-targets of DElncRNAs, and the GO and KEGG analyses related to the DEmRNAs and to the *cis*/*trans*-targets of the DElncRNAs after clicking lncRNA/mRNA (Fig. 5b). In the differential expression analysis module, we provide the search function. The users can input the minimum and maximum values of log_2_(fold change) and input the transcript name for rapid research according to their interest. Furthermore, we provide the search and visualization functions in the *cis*/*trans*-targets of DElncRNA pages, which can help users to conveniently view the targets of specific lncRNA (Fig. 5c). In *cis*-targets of the DElncRNA page, all interactions will be shown in tables and network diagrams after inputting the lncRNA name and clicking the search. Differently, in *trans* targets of the DElncRNA page, all interactions will be shown in tables after searching for a specific lncRNA, while only the top 20 interactions based on the correlation coefficients will be shown in the network diagrams. In addition, users can also browse the locations of interested lncRNAs and their *cis*/*trans*-targets by clicking the lncRNA name or partnerRNA transcript/mRNA in the *cis*/*trans*-targets of DElncRNA pages. The RNA-seq data used for the above analysis in each biotic stress could be found in the heatmap images.

#### Download

There are four download buttons in the “Download” page, including “All,” “Browse,” “Expression,” and “Biotic stress” buttons. The users can download the sample, lncRNA/mRNA information, and sequence data by clicking the “Browse” corresponding download button; download the lncRNA/mRNA (raw count) and lncRNA/mRNA (TPM) data by clicking the “Expression” corresponding download button; and download the data of differential expression analyses of lncRNA and mRNA, the *cis*- and *trans*-targets of DElncRNAs, and the GO and KEGG analyses related to the DEmRNAs and to the *cis*/*trans*-targets of the DElncRNAs by clicking the “Biotic stress” corresponding download button. In addition, the users can also conveniently download all the above-mentioned data by clicking the “All” corresponding download button. Therefore, through the “Download” interface, users can easily download the database data for further analysis according to their interests. We only selected RNA-seq data in one condition and related control group for each disease using for differential expression analyses and further function enrichment analysis. For example, three RNA-seq data under conditions infected by *P. infestans* for 2 days and related control group are selected to analyze the DElncRNAs and predict the potential function of lncRNAs in *P. infestans* infections in potatoes. However, there are 138 RNA-seq data related to *P. infestans* infections under different conditions in the database (Supplementary Table S1). Users can download the raw count and TPM values of lncRNA/mRNA at all RNA-seq data and select samples under specific conditions for further analysis according to their requirements.

#### Related Publications by Our Team

Users can conveniently access the EVLncRNAs 3.0 database [31] and EVlncRNA-Dpred [65] built by our team by clicking the corresponding image on the homepage and they can access the article by clicking the article title in this part. EVLncRNAs 3.0 is a database for functional lncRNAs validated by low-throughput experiments, and two stress-related potato lncRNAs are included in the database. EVlncRNA-Dpred is a computational tool to help separate the potentially functional lncRNAs from the high-throughput sequencing-derived lncRNAs. Users can evaluate the potential function of potato lncRNAs of interest in the PotatoBSLnc before conducting further molecular biology experiments to verify their hypothesis. In addition, if the function of lncRNAs in the PotatoBSLnc is validated by users, they can upload the lncRNAs to the EVLncRNAs 3.0 database to help other researchers.

## Discussion

Over the past two decades, great efforts have been made to reveal the functional lncRNAs in plants. In addition, a large amount of plant RNA-seq data have been generated due to the rapid development of high-throughput sequencing techniques. Recently, some plant lncRNA-related databases have been constructed based on the RNA-seq data from the public database, such as PLncDB [27], CANTATAdb [24], PSRN [28], and JustRNA [29]. Among them, no database is specially built to archive potato biotic stresses related to lncRNAs.

The functional roles of lncRNAs in various abiotic stress responses have been well documented. Differently, the role of lncRNAs in controlling plant biotic stresses is an emerging field, and more and more studies come up recently with novel discoveries [66]. Previous studies mainly focused on the function of lncRNAs in response to biotic stress in model plants, such as tomato [13]. To date, only lncRNAs responsive to *P. carotovorum* and *P. infestans* infections in potatoes have been found [2, 22]. For example, the lncRNA *StlncRNA13558* can enhance potato resistance to *P. infestans* infections [23]. However, the potato lncRNAs that might control other biotic stresses are still unclear. Furthermore, there are some biotic stresses related RNA-Seq data of potatoes in the NCBI, GSA, and EBI databases, which provide a solid foundation for annotating potato biotic stresses related lncRNAs. Therefore, there is an urgent need to build a comprehensive database to archive potato lncRNAs response to biotic stresses with the available RNA-seq data in the public database.

Here, we had constructed the PotatoBSLnc, a repository that contains 18 636 lncRNAs based on 364 RNA-seq data derived from 12 kinds of biotic stresses in potatoes. We annotated the lncRNAs by using a standardized pipeline according to the widely used plant lncRNA annotation criteria (Fig. 1a). Like PLncDB, the PotatoBSLnc provides not only basic information for each lncRNA but also the expression information. Then, to help understand the potential function of lncRNAs in response to each of the 12 biotic stresses, we selected the subsets of RNA-seq data related to each stress to identify the DElncRNAs among infected and control samples and performed the GO and KEGG analyses based on the targets of DElncRNAs. We found that some targets of DElncRNAs derived from different disease (such as *P. carotovorum* and *S. subterranea*) infected and control groups were enriched in the phenylpropanoid biosynthesis pathway, which indicated that some lncRNAs might participate in the plant defense to biotic stresses by regulating the phenylpropanoid biosynthesis and other pathways. Previous studies showed that phenylpropanoid metabolism could provide plants with lignin for pathogen infection and other biotic stresses [67]. In addition, we manually collected detailed information about each sample used for RNA-seq from publications, such as treatments and organ types in Browse-Samples sub-menu. Meanwhile, users can access all data in PotatoBSLnc via download ports; therefore, it is convenient for them to download data for further analysis according to their needs.

## Conclusion

In this research work, we collect, analyze, and visualize available potato biotic stress RNA-seq data from the NCBI, GSA, and EBI databases based on the articles in the PubMed to construct the PotatoBSLnc. To our knowledge, PotatoBSLnc is the first comprehensive database to provide expression profiles of lncRNAs and mRNAs in response to various biotic stresses in potatoes by using RNA-seq data. Moreover, for each of the 12 biotic stresses, DElncRNAs and DEmRNAs are analyzed, and the targets of DElncRNAs are also predicted for function exploration. Furthermore, PotatoBSLnc provides a systematic and user-friendly platform for researchers to browse and access all data via simple and interactive webpages and download ports. Therefore, we believe that PotatoBSLnc will be a useful platform and provide a new resource that not only facilitate related researchers gaining better insights into potato lncRNAs in response to biotic stress but also speed up the breeding of resistant varieties based on the molecular methods.

The number of biotic stress-related potato RNA-seq data will continue to increase; therefore, we will use the same standard pipeline to annotate lncRNAs and add them into PotatoBSLnc. PotatoBSLnc will continue to be updated in a regular basis in the future.

## Supplementary Material

baaf015_Supp

## Data Availability

Full datasets are available for download in the PotatoBSLnc database.
